# Unsupervised Machine Learning to Identify High Likelihood of Dementia in Population-Based Surveys: Development and Validation Study

**DOI:** 10.2196/10493

**Published:** 2018-07-09

**Authors:** Laurent Cleret de Langavant, Eleonore Bayen, Kristine Yaffe

**Affiliations:** ^1^ Service de Neurologie Hôpital Henri Mondor Assistance Publique Hôpitaux de Paris Créteil France; ^2^ Département de Neurologie Faculté de Médecine Université de Paris Est Créteil France; ^3^ Neuropsychologie Interventionnelle U955 EQ1 Institut Mondor de Recherche Biomedicale Inserm Créteil France; ^4^ Département d'Études Cognitives École Normale Supérieure PSL Research University Paris France; ^5^ Global Brain Health Institute Memory and Aging Center University of California San Francisco, CA United States; ^6^ Service de Médecine Physique et Réadaptation Hôpital de la Pitié Salpêtrière Assistance Publique Hôpitaux de Paris Paris France; ^7^ Médecine Physique et Réadaptation Faculté de Médecine Sorbonne University Paris France; ^8^ Laboratoire d'Économie et de Gestion des Organisations de Santé Département d'Économie de la Santé Université Paris-Dauphine Paris France; ^9^ Center for Population Brain Health Department of Psychiatry, Neurology and Epidemiology and Biostatistics University of California San Francisco, CA United States

**Keywords:** dementia, cognition disorders, health surveys, electronic health records, diagnosis, unsupervised machine learning, cluster analysis, data mining

## Abstract

**Background:**

Dementia is increasing in prevalence worldwide, yet frequently remains undiagnosed, especially in low- and middle-income countries. Population-based surveys represent an underinvestigated source to identify individuals at risk of dementia.

**Objective:**

The aim is to identify participants with high likelihood of dementia in population-based surveys without the need of the clinical diagnosis of dementia in a subsample.

**Methods:**

Unsupervised machine learning classification (hierarchical clustering on principal components) was developed in the Health and Retirement Study (HRS; 2002-2003, N=18,165 individuals) and validated in the Survey of Health, Ageing and Retirement in Europe (SHARE; 2010-2012, N=58,202 individuals).

**Results:**

Unsupervised machine learning classification identified three clusters in HRS: cluster 1 (n=12,231) without any functional or motor limitations, cluster 2 (N=4841) with walking/climbing limitations, and cluster 3 (N=1093) with both functional and walking/climbing limitations. Comparison of cluster 3 with previously published predicted probabilities of dementia in HRS showed that it identified high likelihood of dementia (probability of dementia >0.95; area under the curve [AUC]=0.91). Removing either cognitive or both cognitive and behavioral measures did not impede accurate classification (AUC=0.91 and AUC=0.90, respectively). Three clusters with similar profiles were identified in SHARE (cluster 1: n=40,223; cluster 2: n=15,644; cluster 3: n=2335). Survival rate of participants from cluster 3 reached 39.2% (n=665 deceased) in HRS and 62.2% (n=811 deceased) in SHARE after a 3.9-year follow-up. Surviving participants from cluster 3 in both cohorts worsened their functional and mobility performance over the same period.

**Conclusions:**

Unsupervised machine learning identifies high likelihood of dementia in population-based surveys, even without cognitive and behavioral measures and without the need of clinical diagnosis of dementia in a subsample of the population. This method could be used to tackle the global challenge of dementia.

## Introduction

The number of cases of dementia is projected to triple by 2050 worldwide, with a steeper increase in Latin America and the Caribbean, Africa, Asia, and Oceania [1]. Up to half of older adults with dementia are not diagnosed in high-income countries [2] and this proportion is thought to be even higher in low- and middle-income countries [3]. For example, 77% of people with dementia may be underdiagnosed in Brazil [4]. Not only is the diagnosis of dementia complex because it usually relies on an extensive evaluation, but it is also costly [5]. As a consequence, epidemiological data related to dementia mainly comes from Western Europe, East Asia, and North America, but it remains scarce for other regions [6].

Population-based surveys of aging may represent an underinvestigated source of information to study dementia and its determinants. In population-based surveys, nonmedical interviewers collect a rich set of sociodemographic, health, and functional information from nonclinical and representative populations. Several population surveys across four continents are modeled according to the same protocol as the Health and Retirement Study (HRS) in the United States, providing the opportunity to compare aging outcomes between different countries [7]. However, with a few exceptions, dementia outcome is not reliably reported in these surveys.

Machine learning algorithms can assess vast numbers of variables in large datasets, looking for combinations to reliably predict outcomes [8]. This is the case for supervised machine learning algorithms, which learn from specific outcomes available in a subset of individuals, such as the clinical diagnosis of dementia, to expand the acquired knowledge to the whole sample, thanks to a statistical model. In a different approach, unsupervised machine learning algorithms correspond to data-driven techniques that automatically learn from the relationships between elementary bits of information associated with each variable of a dataset. Contrary to supervised machine learning, unsupervised machine learning algorithms unbiasedly reveal associations or clusters existing within datasets without any a priori teaching model.

Because any representative sample of an aging population includes a subgroup of persons living with dementia, we expect unsupervised machine learning to automatically discover this subgroup, without the need of the clinical diagnosis of dementia in a subsample of this population. Our objective was to develop a methodology capable to identify individuals with high likelihood of dementia using a specific unsupervised machine learning algorithm, hierarchical clustering on principal components, in a development cohort in the United States—the HRS [9]. We tested the accuracy of this method by comparing the outcome classification with the predicted probabilities of dementia according to a previously computed supervised machine learning model [10] using the same HRS dataset and based on the clinical diagnosis of dementia available for a subset of the HRS cohort in the Aging, Demographics, and Memory Study (ADAMS) [11]. We also explored the impact of removing cognitive and behavioral measures from the classification algorithm. We then applied this methodology to a validation cohort, the Survey of Health, Ageing and Retirement in Europe (SHARE) [12]. In both cohorts, longitudinal follow-up during the two waves following classification was used as an additional method to validate the clinical relevance of the unsupervised machine learning method.

## Methods

### Populations

The HRS is a longitudinal population-based survey exploring the health and economic well-being of adults older than 50 years in the United States done every two years since 1992. The HRS sample is stratified geographically and covers all demographic groups. The respondent is randomly selected from all age-eligible household members. Although baseline interviews are conducted face-to-face, follow-up interviews are done by telephone (until 2004), with the exception of respondents older than age 80 years [9]. We chose cross-sectional data from wave 6 of the HRS (January 2002 to February 2003; 18,165 individuals) as our development cohort.

The SHARE is a longitudinal population-based survey of individuals aged 50 years or older based on the same protocol as the HRS. We chose wave 4 of SHARE (May 2010 to April 2012; 58,202 individuals from 16 countries including Austria, Belgium, Czech Republic, Denmark, Estonia, France, Germany, Hungary, Italy, Netherlands, Poland, Portugal, Slovenia, Spain, Sweden, and Switzerland) as our validation cohort.

### Measures

Harmonized data (ie, with identically defined variables), from both the HRS and SHARE surveys were downloaded from the Gateway to Global Aging platform [7]. We included variables covering the domains of demographics, health, health care utilization, and cognition that were present in both the HRS and SHARE cohorts. Variables related to insurance, income, financial and housing wealth were removed a priori from both cohort datasets in order to develop a classification method applicable whatever the economic context. Two different behavioral scales were used for the evaluation of depression: The Center for Epidemiological Study of Depression scale (CES-D) [13] for HRS and the European Union initiative to compare symptoms of depression scale (EURO-D) [14] for SHARE. A total of 92 variables were selected for the HRS cohort and 91 for SHARE (Multimedia Appendix 1). Variables with more than 33% of the data missing were arbitrarily discarded. The remaining missing values were imputed with the regularized iterative principal component analysis (PCA) algorithm of the missMDA package of R software [15], which allows the imputed values to have no weight on the PCA results. This imputation method is complementary to the unsupervised machine learning algorithm we used for classification. Three different datasets were used for each cohort: one with the previous selected variables, one omitting cognitive measures, and one omitting both cognitive and behavioral measures.

### Unsupervised Machine Learning Classification

We ran an agglomerative hierarchical clustering on the 10 first principal components resulting from PCA of the datasets (FactoMineR package, R software) [16]. Hierarchical clustering on principal components was considered to be the best unsupervised machine learning technique in this context because (1) PCA allows to reduce the number of variables without losing important information, (2) hierarchical clustering is a very stable method compared to other unsupervised machine learning techniques (eg, *k*-means), and (3) it complements the imputation method we used to obtain a dataset without missing values. The optimal number of clusters in each dataset was determined after running the NbClust package of R software [17].

### Predicted Probability of Dementia in the Health and Retirement Study

A subset of HRS (856 adults older than age 70) received in-home clinical assessments of dementia (cognitive, behavioral, and functional status) between August 2001 and December 2003 (approximately 1 year after their HRS evaluation) by a nurse and neuropsychology technician, as part of the ADAMS study. Definitive diagnosis of dementia in the ADAMS sample was assigned by a consensus of clinical experts [11], using international diagnostic criteria for dementia (cognitive or behavioral disorders associated with significant decline in social or occupational functioning) [18]. Hurd and colleagues [10] used HRS data and the clinical diagnosis of dementia from ADAMS to compute predicted probabilities of having dementia 1 year after HRS evaluation based on two ordered probit models [19]. They used variables related to age, education, sex, activities of daily living (ADL), and instrumental activities of daily living (IADL) limitations, cognitive scores from the HRS interview immediately preceding the ADAMS assessment, and changes in ADL and IADL limitations, and in cognitive scores from the two preceding HRS surveys (2 years and 4 years before) in a first model. When the respondents in HRS were not capable to answer questions assessing cognition and behavior, they used items from the Informant Questionnaire on Cognitive Decline in the Elderly [20], consisting of 16 questions that address the respondent’s memory and ability to function independently, to compute a second model. Predicted probabilities of dementia from these models were available for 7574 HRS respondents for 2003 [7]. Here, we define high likelihood of dementia in this HRS sample as a predicted probability of dementia greater than .95.

### Longitudinal Change in the Created Clusters

Because predicted probabilities of dementia based on the ADAMS clinical diagnosis of dementia are only available in the HRS cohort, we also used longitudinal follow-up in both the HRS and SHARE cohorts to prove the clinical relevance of our unsupervised machine learning classification. To examine longitudinal change in mobility and functional limitations for individuals of the clusters created by our unsupervised machine learning method based on cross-sectional data, we merged data from the classification wave 6 of HRS with data from waves 3, 4, 5, 7, and 8. The longitudinal HRS follow-up covers an average 10-year period, with 6.1 years before classification wave and 3.9 years after. Similarly, data from SHARE classification wave 4 were merged with data from waves 1, 2, 5, and 6 (wave 3 corresponding to SHARE Life study was discarded because of its different protocol). The longitudinal SHARE follow-up covers an average 10.5-year period, with 6.6 years before classification wave and 3.9 years after. Only individuals present in both the earliest and the latest waves were included in both longitudinal datasets (N=10,235 individuals in HRS and N=8245 individuals in SHARE from nine countries including Austria, Belgium, Denmark, France, Germany, Italy, Spain, Sweden, and Switzerland).

## Results

### Development Cohort: Health and Retirement Study

**Figure 1:**
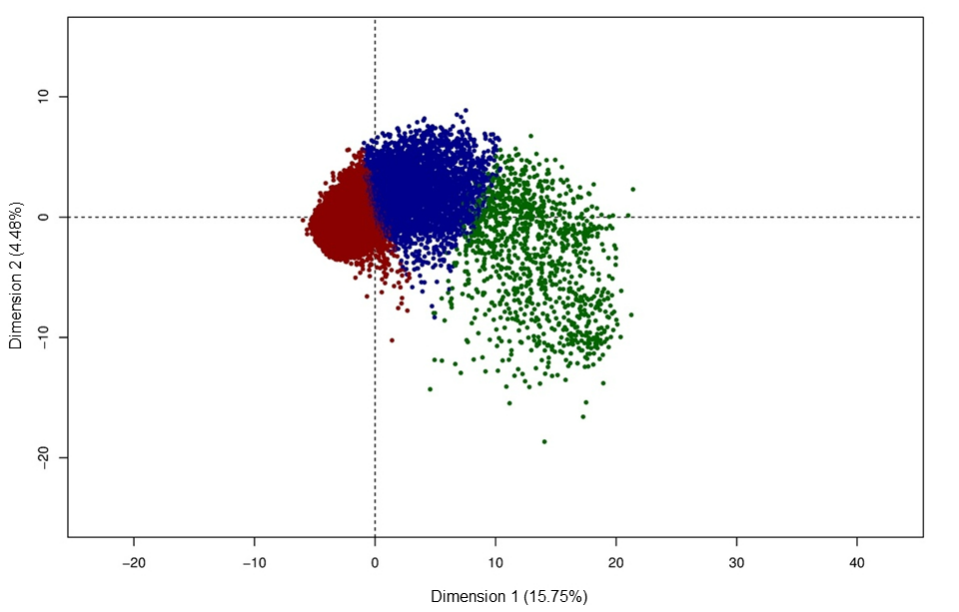
Unsupervised hierarchical clustering in the Health and Retirement Study cohort. Scatterplot of the two first dimensions of the principal component analysis (dimension 1 and dimension 2 with explained variance) for individuals in the three clusters (red=cluster 1, blue=cluster 2, green=cluster 3).

We identified three clusters after running unsupervised machine learning classification (Figure 1). Cluster 1 (n=12,231) corresponds to individuals without any functional impairment on both IADL and ADL, and without significant mobility limitation for climbing stairs or walking. Cluster 2 (n=4841) shows moderate mobility limitations, but no functional impairment. Cluster 3 (n=1093) includes individuals with significant functional impairment and mobility limitations. Compared to clusters 1 and 2, individuals in cluster 3 were older, more often women, more often black or Hispanic, less educated, with poorer memory performance, and less likely to be working (Table 1). The percentage of missing values for cognitive variables before imputation differs in the three clusters: 7.30% (893/12,231) in cluster 1, 10.39% (503/4841) in cluster 2, and 59.10% (646/1093) in cluster 3 (*P*<.001). Similarly, missing values for behavioral measures of depression before imputation amounted to 7.30% (893/12,231) in cluster 1, 10.39% (503/4841) in cluster 2, and 59.01% (645/1093) in cluster 3 (*P*<.001).

Cluster 3 showed 89.5% sensitivity, 93.3% specificity, 93.1% accuracy overall, and an area under the curve (AUC) of 0.91 for dementia compared to the high likelihood of dementia defined as a predicted probability of dementia >.95 (see Table 2 for other thresholds and for comparison to ADAMS). When cognitive measures were removed from the dataset, classification into cluster 3 showed 88% sensitivity, 93.4% specificity, 93.1% accuracy, and an AUC of 0.91.

 When both cognitive and behavioral variables were removed, classification into cluster 3 reached 87% sensitivity, 93.6% specificity, 93.3% accuracy, and an AUC of 0.90. A 98.2% concordant accuracy was found when classifications with and without cognitive variables were compared to each other. The concordant accuracy reached 94.4% between classifications with and without both cognitive and behavioral variables.

**Figure 2:**
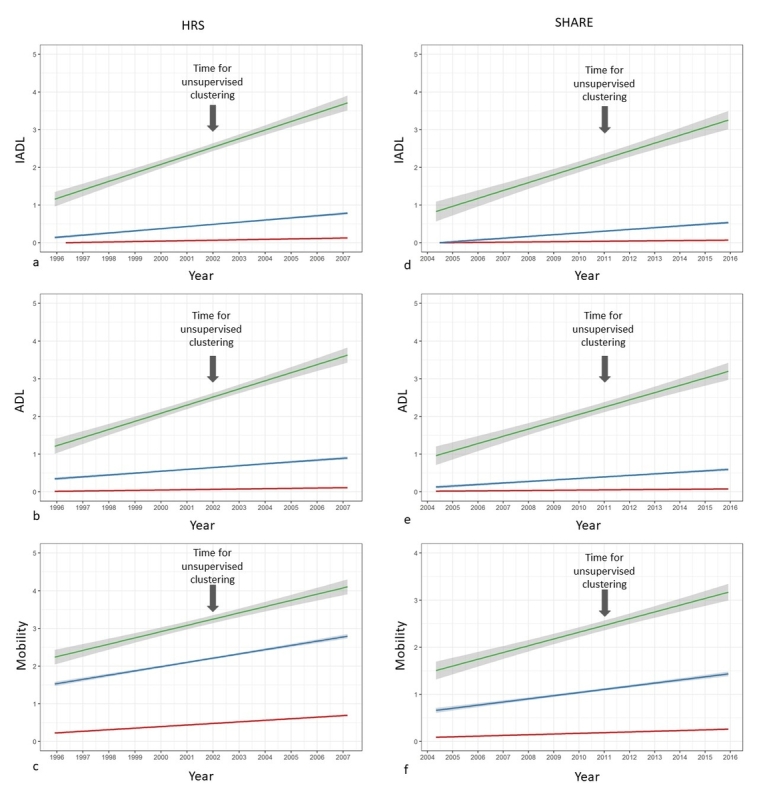
Longitudinal change of instrumental activities of daily living (IADL), activities of daily living (ADL), and mobility scores in both Health and Retirement Study (HRS) and Survey of Health, Ageing and Retirement in Europe (SHARE) cohorts. Linear models with date of assessment at each wave as an independent variable were used to depict the longitudinal change of IADL, ADL, and mobility scores in the three clusters (red=cluster 1, blue=cluster 2, green=cluster 3) in both HRS (left) and SHARE (right) cohorts. A 99% confidence interval (gray color) is drawn for each cluster. The year corresponding to the time of classification is indicated by an arrow.

Among the 18,165 individuals present at the time of classification (wave 6), 14,670 individuals remained present at wave 8, 1152 individuals were dropouts, and 2343 were deceased. The three clusters differed regarding their survival rate after a 3.9-year period following classification. Survival rate was 94.2% (n=715 deceased) in cluster 1, 80.1% (n=963 deceased) in cluster 2, and 39.2% (n=665 deceased) in cluster 3 (*P*<.001). Longitudinal change in mobility and functional limitations for the surviving individuals also differed across the three clusters (Figure 2). Individuals in cluster 1 remained stable during the 10-year period of follow-up (from 6 years before classification wave to 4 years after) according to ADL, IADL, and mobility scores. Individuals in cluster 2 showed mild worsening of ADL and IADL scores and moderate worsening of mobility scores during the same 10-year period. Individuals in cluster 3 showed the steepest worsening of ADL, IADL, and mobility scores during the same period.

### Validation Cohort: Survey of Health, Ageing and Retirement in Europe

**Figure 3:**
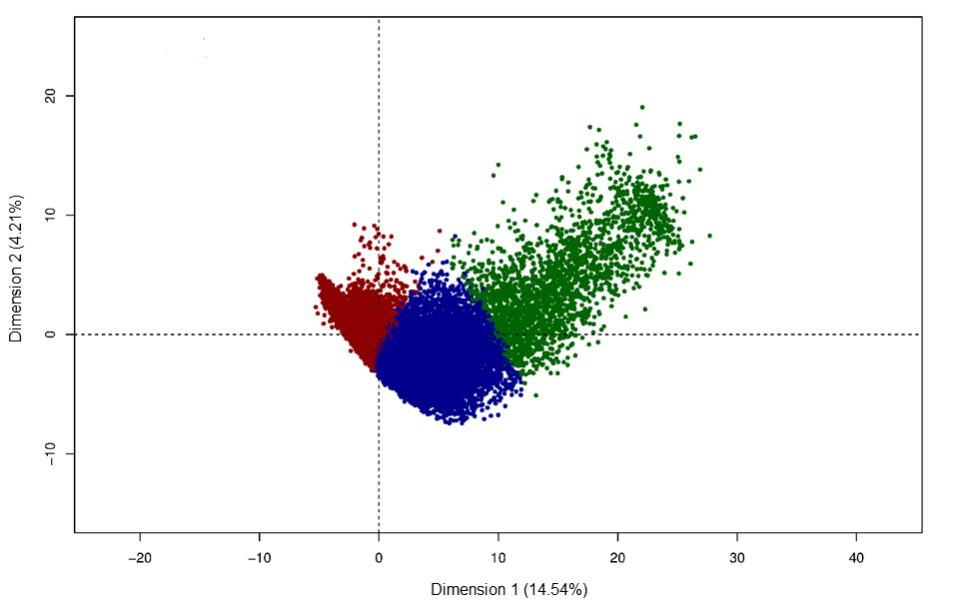
Unsupervised hierarchical clustering in the Survey of Health, Ageing and Retirement in Europe cohort. Scatterplot of the two first dimensions of the principal component analysis (dimension 1 and dimension 2 with explained variance) for individuals in the three clusters (red=cluster 1, blue=cluster 2, green=cluster 3).

The three clusters, cluster 1 (N=40,223), cluster 2 (N=15,644), and cluster 3 (N=2335), identified in the SHARE dataset (Figure 3) showed similar characteristics compared to those identified for HRS (Table 3). Individuals in cluster 3 were older, more often women, less educated, and less likely to be working compared to clusters 1 and 2. The percentage of missing values for cognitive variables before imputation differed in the three clusters: 1.97% (791/40,223) in cluster 1, 2.85% (446/15,644) in cluster 2, and 18.67% (436/2335) in cluster 3 (*P*<.001). Missing values for behavioral measures of depression before imputation reached 1.29% (518/40,223) in cluster 1, 1.89% (296/15,644) in cluster 2, and 15.53% (363/2335) in cluster 3 (*P*<.001). Similar clusters were created if cognitive and behavioral variables were removed from the datasets (96.4% accuracy between classifications with and without cognitive variables; 94.3% accuracy between classifications with and without both cognitive and behavioral variables).

Among the 58,202 individuals present at the time of classification (wave 4), 32,325 were interviewed again at wave 6, whereas 22,406 individuals were dropouts (5861 of those lived in a country not assessed during wave 6) and 3471 were deceased. After a 3.9-year period following classification, the survival rate was 97.1% (n=1067 deceased) in cluster 1, 88.6% (n=1593 deceased) in cluster 2, and 62.2% (n=811 deceased) in cluster 3 (*P*<.001). The surviving individuals in cluster 1 remained stable during a 10.5-year period (6 years before classification wave to 4 years after), whereas those from cluster 2 showed moderate mobility decline and very mild functional decline. The surviving individuals from cluster 3 showed progressive loss of autonomy according to ADL and IADL scores, and severe mobility impairment over the same period (Figure 2).

**Table 1:** Demographic and clinical characteristics in the three clusters created by unsupervised machine learning in the Health and Retirement Study (HRS) cohort.

Demographic and clinical characteristics		All (N=18,165)	Cluster 1 (n=12,231)	Cluster 2 (n=4841)	Cluster 3 (n=1093)	*P* value^a^
Age (years), mean (SD)		68.4 (10.5)	66.1 (9.5)	71.4 (10.4)	79.7 (11.3)	<.001
Gender (male), n (%)		7456 (41.05)	5580 (45.62)	1510 (31.19)	366 (33.49)	<.001
Education (years), mean (SD)		12.1 (3.4)	12.8 (3.0)	10.9 (3.5)	10 (4.0)	<.001
**Race/ethnicity, n (%)**						
	White	14,967 (82.39)	10,373 (84.81)	3770 (77.87)	824 (75.39)	<.001
	Black	2508 (13.81)	1423 (11.63)	866 (17.89)	219 (20.04)	<.001
	Hispanic	1472 (8.10)	886 (7.24)	465 (9.61)	121 (11.07)	<.001
	Other race/ethnicity	685 (3.77)	434 (3.55)	201 (4.15)	50 (4.57)	.07
Working full time, n (%)		3773 (20.77)	3470 (28.37)	301 (6.22)	2 (0.18)	<.001
**Functional characteristics, mean (SD)**						
	IADL^b^ (0-5)	0.4 (1.0)	0 (0.2)	0.5 (0.8)	3.6 (1.4)	<.001
	ADL^c^ (0-5)	0.4 (1.0)	0 (0.1)	0.6 (0.9)	3.5 (1.4)	<.001
	Mobility^d^ (0-5)	1.2 (1.5)	0.4 (0.7)	2.5 (1.4)	3.9 (1.4)	<.001
	Total word recall (0-20)	9.4 (4.1)	10.5 (3.4)	8.4 (3.5)	2.2 (4.2)	<.001
	CES-D^e^ (0-8)	1.6 (2.1)	0.9 (1.3)	3.2 (2.2)	3.4 (3.2)	<.001
**Clinical characteristics, n (%)**						
	Ever had high blood pressure	9167 (50.47)	5265 (43.05)	3183 (65.75)	719 (65.78)	<.001
	Ever had diabetes	3029 (16.67)	1456 (11.90)	1286 (26.56)	287 (26.26)	<.001
	Ever had cancer	2337 (12.87)	1364 (11.15)	788 (16.28)	185 (16.93)	<.001
	Ever had lung disease	1473 (8.11)	499 (4.08)	801 (16.57)	173 (15.83)	<.001
	Ever had heart disease	4219 (23.23)	1854 (15.16)	1843 (38.07)	521 (47.67)	<.001
	Ever had stroke	1567 (8.63)	469 (3.83)	654 (13.51)	444 (40.62)	<.001
	Ever had arthritis	10,231 (56.32)	5501 (44,98)	3903 (80.62)	826 (75.57)	<.001
	Ever smoked	10,623 (58.48)	7105 (58.09)	2954 (61,02)	564 (51.60)	<.001
	Ever drank alcohol	8103 (44.61)	6573 (53.74)	1410 (29.13)	120 (10,98)	<.001
	Body mass index, mean (SD)	27.2 (5.4)	26.9 (4.7)	28.3 (6.5)	25.2 (6.4)	<.001

^a^*P* values are from one-way analysis of variance (ANOVA) or chi-square tests as appropriate.

^b^IADL: instrumental activities of daily living, including any difficulty using a telephone, taking medication, handling money, shopping, and preparing meals.

^c^ADL: activities of daily living, including any difficulty bathing, eating, dressing, walking across a room, and getting in or out of bed.

^d^Mobility: any difficulty for walking several blocks, walking one block, walking across the room, climbing several flights of stairs and climbing one flight of stairs.

^e^CES-D: Center for Epidemiological Study of Depression Scale [13].

**Table 2:** Classification performance of cluster 3 from unsupervised machine learning in the Health and Retirement Study (HRS) cohort compared to various thresholds of predicted probabilities of dementia from Hurd et al’s model and to the Aging, Demographics, and Memory Study (ADAMS) clinical diagnosis of dementia.

Classification performance of cluster 3	Predicted probability of dementia^a^ (N=7574)				ADAMS clinical diagnosis of dementia (N=834)
	>.50	>.75	>.90	>.95	
Sensitivity (%)	62.9	77.3	86.7	89.5	59.3
Specificity (%)	96.4	95.1	94.2	93.3	93.0
Accuracy (%)	92.1	93.6	93.7	93.1	81.3

^a^Hurd et al’s model [10].

**Table 3:** Demographic and clinical characteristics in the three clusters created by unsupervised machine learning in the Survey of Health, Ageing and Retirement in Europe (SHARE) cohort.

Demographic and clinical characteristics		All (N=58,202)	Cluster 1 (n=40,223)	Cluster 2 (n=15,644)	Cluster 3 (n=2335)	*P* value^a^
Age (years), mean (SD)		65.4 (10.4)	62.7 (9.0)	70.7 (10.2)	77.4 (10.6)	<.001
Gender (male), n (%)		25,182 (43.26)	19,469 (48.40)	4825 (30.84)	888 (38.03)	<.001
Education (years), mean (SD)		10.3 (4.3)	11.1 (4.1)	8.8 (4.0)	7.8 (4.3)	<.001
Working, n (%)		3889 (6.68)	3591 (8.93)	281 (1.80)	17 (0.73)	<.001
**Functional characteristics, mean (SD)**						
	IADL^b^ (0-5)	0.2 (0.8)	0 (0.2)	0.3 (0.6)	3.1 (1.5)	<.001
	ADL^c^ (0-5)	0.2 (0.8)	0 (0.1)	0.4 (0.7)	3.2 (1.4)	<.001
	Mobility^d^ (0-4)	0.6 (1.0)	0.1 (0.4)	1.3 (1.0)	3.1 (1.1)	<.001
	Total word recall (0-20)	8.9 (3.8)	9.9 (3.4)	7 (3.5)	3.7 (3.8)	<.001
	EURO-D^e^ (0-12)	2.6 (2.3)	1.8 (1.7)	4.3 (2.4)	5.1 (2.8)	<.001
**Clinical characteristics, n (%)**						
	Ever had high blood pressure	22,848 (39.26)	12,840 (31.92)	8846 (56.55)	1162 (49.76)	<.001
	Ever had diabetes	7208 (12.38)	3136 (7.78)	3481 (22.25)	591 (25.31)	<.001
	Ever had cancer	3076 (5.29)	1510 (3.75)	1357 (8.67)	209 (8.95)	<.001
	Ever had lung disease	3835 (6.59)	1444 (3.59)	2051 (13.11)	340 (14.56)	<.001
	Ever had heart disease	7999 (13.74)	2975 (7.40)	4249 (27.16)	775 (33.19)	<.001
	Ever had stroke	2547 (4.37)	638 (1.59)	1286 (8.22)	623 (26.68)	<.001
	Ever had arthritis	14,192 (24.38)	5797 (14.41)	7347 (46.96)	1035 (44.33)	<.001
	Ever smoked	27,097 (46.56)	20,120 (50.02)	6163 (39.40)	814 (34.86)	<.001
	Ever drank alcohol	45,893 (78.85)	34,061 (84.68)	10,620 (67.89)	1212 (51.91)	<.001
	Body mass index, mean (SD)	26.9 (4.8)	26.4 (4.2)	28.2 (5.9)	26.9 (5.8)	<.001

^a^*P* values are from one-way ANOVAs or chi-square tests as appropriate.

^b^IADL: instrumental activities of daily living, including any difficulty using a telephone, taking medication, handling money, shopping, and preparing meals.

^c^ADL: activities of daily living, including any difficulty bathing, eating, dressing, walking across a room, and getting in or out of bed.

^d^Mobility: any difficulty for walking 100 meters, walking across a room, climbing one flight of stairs, and climbing several flights of stairs.

^e^EURO-D: European Union initiative to compare symptoms of depression scale [14].

## Discussion

We used unsupervised machine learning and cross-sectional data from two population-based surveys in the United States and Europe to identify individuals with high likelihood of dementia. Although the clinical diagnosis of dementia usually requires a lengthy and costly process based on human expertise and clinical data, we show that unsupervised machine learning applied to data from population-based surveys provides an accurate estimation of the high probability of dementia, even in the absence of cognitive or behavioral variables. The impact of using unsupervised machine learning in nonmedical datasets would serve to identify older adults with high likelihood of dementia. Being classified into cluster 3 according to our unsupervised machine learning method has clear clinical implications, as shown by the low survival rate during follow-up and the steep functional and mobility declines in the surviving individuals in both the HRS and SHARE cohorts. The higher death rate observed in HRS in comparison to SHARE is likely explained by the older age of the HRS cohort, the better reporting of death date in HRS because of the National Death Index, and the higher number of dropouts in SHARE. Because this unsupervised machine learning method identifies the individuals with worse clinical outcomes, it would be valuable to target those individuals and offer them care including close follow-up or even referral for trials.

Although supervised machine learning is being increasingly used to predict dementia based on clinical data, this study is the first to use unsupervised machine learning and nonclinical data from population-based surveys to identify subjects at risk of dementia. Yet, unsupervised machine learning may be difficult to understand from a clinical perspective. Certain authors compare this purely data-driven method to a “black box” in which the actual mechanisms leading to the outcome remain opaque [21]. In fact, these unsupervised techniques also bring advantages. Because they do not rely on a prespecified clinical outcome (eg, the diagnosis of dementia in a subsample of the population), they are more flexible than supervised machine learning models and they can be more easily transferred to different types of datasets. Here, this allows classification of individuals from the SHARE cohort where clinical diagnosis of dementia is not available. Moreover, because the unsupervised machine learning algorithm we used is based on PCA, it can assess many variables, such as educational level [22], decline in physical activity [23], slowing gait [24], clinical comorbidities, alcohol consumption, smoking, and weight variations [25], or health care use [26], which are known to be important in the context of dementia. This unsupervised machine learning technique also demonstrates that removing cognitive and behavioral measures from the datasets does not significantly impact the accuracy of the classifications in both HRS and SHARE. The latter result was unexpected given that the current diagnosis criteria of dementia heavily relies on cognitive and behavioral measures [18]. Presumably, this unsupervised machine learning technique is capable of identifying participants with significant decline in social or occupational functioning, often associated with cognitive and behavioral disorders in the context of dementia. It may be of interest for clinicians and researchers because it could allow them to use datasets lacking cognitive or behavioral information such as electronic medical records (EMRs) for studying dementia.

Several aspects of the unsupervised machine learning classification we used may allow for its wide application. In both the HRS and SHARE cohorts, cluster 3 identifies participants who are older; with more cognitive, motor, and functional difficulties; and more likely to show further decline and higher death rate. Thus, this unsupervised machine learning classification technique could be used in other population-based surveys of the HRS family lacking a clinical assessment of dementia such as in ADAMS for the HRS cohort. The longitudinal HRS family studies include the Mexican Health and Aging Study, the English Longitudinal Study of Ageing, the Costa Rican Longevity and Healthy Aging Study, the Korean Longitudinal Study of Aging, the Indonesian Family Life Survey, the Japanese Study of Aging and Retirement, the Asian-African Study on Global AGEing and Adult Health, the Irish Longitudinal Study on Ageing, the Chinese Health and Retirement Longitudinal Study, and the Longitudinal Aging Study in India [7]. Moreover, this unsupervised machine learning algorithm uses cross-sectional data, thus allowing classification of a larger sample of participants at each time point of the survey than the sample that would be constituted if longitudinal data were required. This explains why our method can classify the whole cohort of HRS (N=18,165) compared to the smaller sample (N=7574) in Hurd et al’s model [10]. Omitting both cognitive and behavioral variables might further facilitate the inclusion of a larger number of individuals in population surveys. Finally, because it is efficient in two different populations in the United States and Europe even without cognitive or behavioral measures, we expect this classification method to be applicable in other datasets if they constitute representative samples of an aging population.

A possible limitation in this study could be the chosen gold standard to test the accuracy of our classification in HRS cohort. The predicted probabilities of dementia from Hurd et al’s models [10] constitute the best reference standard available but they are not definitive. Importantly, Hurd and colleagues provide predicted probabilities of having dementia 1 year after HRS evaluation, whereas our unsupervised machine learning classification directly applies for the time of evaluation, which might account for discrepancies between the two methods. In addition, because Hurd and colleagues used two different models, one when respondents provided answers to cognitive and behavioral measures and another when proxies provided these answers, this might constitute a bias in their predicted probabilities of dementia. The .95 threshold we used for predicted probability of dementia according to Hurd et al’s model [10] undoubtedly identifies subjects with high likelihood of dementia, but also misses actual cases of dementia with lower predicted probabilities according to the same model. Indeed, using either lower thresholds of predicted probability of dementia or the actual clinical diagnosis in the smaller sample from ADAMS [11], we obtain similar specificity, but lower sensitivity of cluster 3 regarding the likelihood of dementia (Table 2). Noteworthy, even the diagnosis of dementia from ADAMS and thus the derived Hurd et al’s model might suffer from a classification error bias like any clinical assessments [27]. This is why we also use follow-up information related to survival rate and to longitudinal change in functional and mobility scores in the three clusters created after unsupervised machine learning as another way to check for face validity. As expected from patients with dementia, the individuals classified into cluster 3 show a low survival rate and a progressive decline beginning years before the classification time point. Altogether, we acknowledge that this classification method cannot be considered as a diagnosis tool for dementia, or even a dementia-screening instrument, given its moderate sensitivity. Yet, the outcome of this classification, cluster 3, still offers opportunities for new medical applications and new avenues of research in the field of dementia.

Our method could be applied to tackle global health estimates of dementia burden. For example, using the HRS family studies, it could provide a global estimate of dementia across four different continents and an unprecedented cross-country comparison of its socioeconomic consequences, determinants, and risk factors. It could also be applied to other population-based surveys based on different protocols or even to EMRs, often lacking cognitive or behavioral measurements. Whether or not and how the participants at risk of having dementia should be informed after unsupervised machine learning classification raises an ethical issue that would require a large debate. After further validation and using more parsimonious datasets, we expect this unsupervised machine learning classification to impact clinical practice in resource-poor areas with limited primary care access and limited cognitive testing capacities. This technique could support, but not replace, human expertise [28] by identifying groups of individuals with high likelihood of dementia who could then get further clinical assessment and care. Unsupervised machine learning classification applied to existing population datasets or EMRs may help prepare for the global challenge of dementia.

## Appendix

Multimedia Appendix 1 Variables used in the Health and Retirement Study (HRS) and Survey of Health, Ageing and Retirement in Europe (SHARE) datasets. Asterisk indicates variables related to the past year in SHARE, to the 2 past years in HRS; § to those omitted in datasets without cognitive variables; and # to those omitted in the datasets without behavioral variables.

## Abbreviations

ADAMS: Aging, Demographics, and Memory Study
ADL: activities of daily living
ANOVA: analysis of variance
AUC: area under the curve
CES-D: Center for Epidemiological Study of Depression Scale
EMR: electronic medical record
EURO-D: European Union initiative to compare symptoms of depression scale
HRS: Health and Retirement Study
IADL: instrumental activities of daily living
PCA: principal component analysis
SHARE: Survey of Health, Ageing and Retirement in Europe
