# Supplementary material for: Unsupervised Machine Learning to Identify High Likelihood of Dementia in Population-Based Surveys: Development and Validation Study
Source: J Med Internet Res. 2018 Jul 9;20(7):e10493. doi: 10.2196/10493 (PMC6056741; doi:10.2196/10493)
Supplement: Multimedia Appendix 1 [file jmir_v20i7e10493_app1.pdf]

	HRS Wave 6 (N=18165)		SHARE Wave 4 (N=58202)	
	Variable name	N missing	Variable name	N missing
**Demographics**				
Whether Proxy Interview	r6proxy	0	r4proxy	244
Age (years) at Interview	r6agey_m	0	r4agey	7
Gender	ragender	0	ragender	0
Census Region	r6cenreg	30	-	-
Census Division	r6cendiv	16	-	-
Country code	-	-	isocountry	0
Years of Education	raedyrs	5	raedyrs	283
Marital Status	r6mstat	15	r4mstat	2237
Place of Birth (Cens Region)	rabplace	21	rabplace	962
**Labor force**				
Labor Force Status	r6lbrf	0	r4lbrf_s	1
**Family**				
Mother alive	r6momliv	378	r4momliv	16132
Father alive	r6dadliv	240	r4dadliv	17515
Number of living parents	r6livpar	479	r4livpar	18847
Mother age current/at death	r6momage	790	r4momage	22490
Father age current/at death	r6dadage	953	r4dadage	20480
Number of living children	h6child	230	h4child	0
Number of living brothers	r6livbro	29	r4livbro	6869
Number of living sisters	r6livsis	34	r4livsis	6873
Number of living siblings	r6livsib	50	r4livsib	6907
**Health**				
Self-report of health	r6shlt	11	r4shlt	303
Health problems limit work	r6hlthlm	96	r4hlthlm_s	288
Self-rated memory	r6slfmem	2036	r4slfmem	1116
Some Difficulty-Walk across room	r6walkra	22	r4walkra	319
Some Difficulty-Dressing	r6dressa	13	r4dressa	319
Some Difficulty-Bathing, shower	r6batha	14	r4batha	319
Some Difficulty-Eating	r6eata	21	r4eata	319
Some Difficulty-Get in/out bed	r6beda	28	r4beda	319
Some Difficulty-Using the toilet	r6toilta	37	r4toilta	319
Some Difficulty-Use a map	r6mapa	2408	r4mapa	319
Some Difficulty-Use telephone	r6phonea	105	r4phonea	319
Some Difficulty-Managing money	r6moneya	593	r4moneya	319
Some Difficulty-Take medications	r6medsa	396	r4medsa	319
Some Difficulty-Shop for grocery	r6shopa	658	r4shopa	319
Some Difficulty-Prepare hot meal	r6mealsa	1050	r4mealsa	319
Some Difficulty-Walk sev blocks	r6walksa	472	-	-
Some Difficulty-Walk one block	r6walk1a	200	-	-
Some difficulty-walk 100m	-	-	r4walk100a	315
Some Difficulty-Sit for 2 hours	r6sita	187	r4sita	315
Some Difficulty-Get up fr chair	r6chaira	50	r4chaira	315
Some Difficulty-Clmb sev flt str	r6climsa	2033	-	315
Some Difficulty-Clmb 1 flt stair	r6clim1a	657	r4clim1a	315
Some Difficulty-Stoop/Kneel/Crch	r6stoopa	318	r4stoopa	315
Some Difficulty-Lift/carry 10lbs	r6lifta	801	r4lifta	315
Some Difficulty-Pick up a dime	r6dimea	66	r4dimea	315
Some Difficulty-Rch/xtnd arms up	r6armsa	102	r4armsa	315
Some Difficulty-Push/pull lg obj	r6pusha	1347	r4pusha	315
Some Difficulty-ADLs /0-5	r6adla	7	r4adla	319
Some Difficulty-ADLsWallace /0-3	r6adlwa	8	r4adlwa	319
Some Difficulty-IADLs /0-3	r6iadla	9	r4iadla	319

Some Difficulty-IADLs /0-5	r6iadlza	8	r4iadlza	319
Some Difficulty-Mobility /0-5	r6mobila	14	-	-
Some Difficulty-mobility /0-4	-	-	r4mobilb	302
Some Diff-Large Muscle /0-4	r6lgmusa	14	r4lgmusa	315
Walk1/R,Clim1,Bed,Bath/0-5	r6grossa	7	r4grossa	302
Dime/Eat/Dress /0-3	r6finea	8	r4finea	302
**Health care use**				
Hospital stay	r6hosp	44	r4hosp1y*	417
# Hospital stays	r6hsptim	88	r4hsptim1y*	445
# Nights in hospital	r6hspnit	167	r4hspnit1y*	483
Nursing home stay	r6nrshom	28	r4nrshom1y*	804
# Nursing home stays	r6nrstim	58	r4nrstim1y*	954
# Nights in nursing home	r6nrsnit	68	r4nrsnit1y*	809
Live in Nursing home at Interview	r6nhmliv	0	r4nhmliv1y*	804
Doctor visit	r6doctor	22	r4doctor1y*	778
# Doctor visits	r6doctim	714	r4doctim1y*	778
**Chronic conditions and life style**				
Ever had high blood pressure	r6hibpe	22	r4hibpe	318
Ever had diabetes	r6diabe	22	r4diabe	318
Ever had cancer	r6cancre	32	r4cancre	318
Ever had lung disease	r6lunge	20	r4lunge	318
Ever had heart problems	r6hearte	19	r4hearte	318
Ever had stroke	r6stroke	16	r4stroke	318
Ever had arthritis	r6arthre	29	r4arthre	318
Body Mass Index=kg/m2	r6bmi	347	r4bmi	3072
Height in meters	r6height	35	r4height	2276
Weight in kilograms	r6weight	316	r4weight	1221
Whether vigorous phys act 3+/wk	r6vigact	7	r4vgactx*	734
Ever drinks any alcohol	r6drink	4	r4drink	713
# days/week drinks	r6drinkd	36	-	-
Frequency of drinking	-	-	r4drinkx	728
Ever smoked	r6smokev	179	r4smokev	2305
Smokes now	r6smoken	1	r4smoken	712
**Cognition**				
Immediate word recall	r6imrc §	2036	r4imrc §	1590
Delayed word recall	r6dlrc §	2036	r4dlrc §	1593
Serial 7s	r6ser7 §	2036	r4ser7 §	3867
Cognition Date naming-Month	r6mo §	8554	r4mo §	22049
Cognition Date naming-Day of month	r6dy §	8554	r4dy §	22108
Cognition Date naming-Year	r6yr §	8554	r4yr §	22055
Cognition Date naming-Day of week	r6dw §	8554	r4dw §	22054
Total word recall summary score	r6tr20 §	2036	r4tr20 §	1673
**Behavior**				
CESD Felt depressed	r6depres #	2055	-	-
CESD Everything an effort	r6effort #	2055	-	-
CESD Sleep was restless	r6sleepr #	2057	-	-
CESD Was happy	r6whappy #	2071	-	-
CESD Felt lonely	r6flone #	2057	-	-
CESD Felt sad	r6fsad #	2058	-	-
CESD Could not get going	r6going #	2062	-	-
CESD Enjoyed life	r6enlife #	2064	-	-
EUROD depression	-		r4depress #	1136
EUROD pessimism	-		r4pessim #	1291
EUROD suicidality	-		r4suicid #	1290
EUROD guilt	-		r4guilt #	1289

EUROD sleep	-		r4sleep #	1094
EUROD interest	-		r4intrst #	1162
EUROD irritability	-		r4irritb #	1165
EUROD appetite	-		r4appett #	986
EUROD fatigue	-		r4fatig #	1158
EUROD concentration	-		r4concnt #	1235
EUROD enjoyment	-		r4enjoym #	1177
EUROD tearfulness	-		r4tearfl #	1182
